# The screening value of mammography for breast cancer: an overview of 28 systematic reviews with evidence mapping

**DOI:** 10.1007/s00432-025-06122-z

**Published:** 2025-03-06

**Authors:** Jiyuan Shi, Jiang Li, Ya Gao, Wanqing Chen, Liang Zhao, Ni Li, Jinhui Tian, Zheng Li

**Affiliations:** 1 School of Nursing, Beijing University of Chinese Medicine, Beijing, 100144 China; 2 National Cancer Center, National Clinical Research Center for Cancer; Cancer Hospital, Chinese Academy of Medical Sciences and Peking Union Medical College, Beijing, 100021 China; 3 Evidence-Based Medicine Center, Lanzhou University, Lanzhou, 730000 China; 4 Key Laboratory of Evidence-Based Medicine and Knowledge Translation of Gansu Province, Lanzhou University, Lanzhou, 730000 China; 5 School of Nursing, Chinese Academy of Medical Sciences & Peking Union Medical College, Beijing, China

**Keywords:** Breast cancer, Mammography, Screening, Meta-analysis, Evidence mapping

## Abstract

**Background:**

The effectiveness of mammography screening in reducing breast cancer mortality and the accuracy of various mammography techniques have been widely studied. However, the quality and findings of existing systematic reviews and meta-analyses require comprehensive evaluation.

**Methods:**

A systematic literature search was conducted in the Cochrane Library, EMBASE, and PubMed for systematic reviews published up until December 20, 2022. A total of 28 systematic reviews with meta-analyses were included. Two reviewers independently extracted data and assessed methodological quality using the Risk Of Bias In Systematic Reviews (ROBIS) tool.

**Results:**

Of the 28 systematic reviews included, only 17.9% were rated as low risk of bias. The pooled estimates for breast cancer mortality reduction due to mammography screening ranged from 0.51 (95% CI 0.46–0.55) to 1.04 (95% CI 0.84–1.27). The results were influenced by study design, age, and follow-up duration, with an overall trend indicating that mammography screening reduces breast cancer mortality. Sensitivity of mammography techniques ranged from 55 to 91%, and specificity from 84 to 97%. Digital breast tomosynthesis combined with synthetic contrast-enhanced spectral mammography, digital mammography, and film mammography demonstrated relatively high cancer detection rates and low false positives.

**Conclusion:**

Mammography screening appears effective in reducing breast cancer mortality. The accuracy of various mammography techniques is generally reliable, with certain combinations showing high detection rates. However, the methodological quality of most included reviews was at high risk of bias, indicating a need for higher-quality studies in the future.

**Supplementary Information:**

The online version contains supplementary material available at 10.1007/s00432-025-06122-z.

## Introduction

Breast cancer is the most commonly diagnosed cancer and the leading cause of cancer death among women, nearly 2.1 million new cases of breast cancer and 630000 cancer deaths owing to breast cancer were recorded worldwide in 2018 (Bray et al. 2018). Early detection of breast cancer through screening, along with effective diagnostic pathways and optimal treatment, has the potential to reduce breast cancer mortality rates and alleviate the burden of the disease on the population. Over the past few decades, the mortality rate of breast cancer has significantly decreased, likely due to improvements in screening rates and advances in screening technologies (Nelson et al. 2009).

Screening accuracy is crucial, as false positives and negatives can impact early detection, treatment outcomes, and patient psychology. False positives may cause unnecessary anxiety and procedures, while false negatives can delay diagnosis and worsen prognosis. Therefore, researchers are still continuously working to refine screening technologies to improve accuracy and enhance the overall effectiveness of breast cancer detection (Nelson et al. 2009; Yang et al. 2020; DeSantis et al. 2013, 2011). A variety of screening methods have been used for breast cancer screening among the general female population including breast self-examination, clinical breast examination (CBE), ultrasonography, and mammography (Yang et al. 2020). Mammography has been recognized as an effective way to aid in the detection of breast cancer at an earlier stage, which may be associated with breast cancer mortality reduction (DeSantis et al. 2013, 2011; Myers et al. 2015). Numerous systematic reviews have examined that screening with mammography reduces breast cancer mortality among women, however, the result of breast cancer mortality reduction was inconsistent and insufficient evidence was found for variation of effectiveness of mammography by age (Myers et al. 2015; Brodersen et al. 2010; McInnes et al. 2018).

In recent years, there have been significant efforts to enhance the performance of mammography screening through the adoption of new technologies, such as digital mammography (DM), digital breast tomosynthesis (DBT), contrast-enhanced energy spectrum mammography (CESM), and digital breast tomosynthesis plus full-field digital mammography (FFDM). Recently, some systematic reviews have compared the performance of different imaging techniques for breast cancer screening, the overall results have remained mixed or inconclusive (Nothacker et al. 2009; Movik et al. 2017), and the methodological quality of these systematic reviews has not been evaluated.

The methodological quality of systematic reviews is crucial for ensuring the reliability of synthesized evidence used in clinical practice and policy decisions. Several tools have been developed to assess the methodological quality of systematic reviews, with ROBIS being specifically designed to evaluate the risk of bias. ROBIS is applicable to a wide range of systematic reviews, including those focused- on interventions, diagnostics, etiology, and prognosis, and it can also assess the relevance of the review questions to practical issues (Muchadeyi et al. 2024; Perry et al. 2017; Whiting et al. 2016).

Given the mixed or inconclusive previous review outcomes and the lack of robust methodological quality appraisal of the existing systematic reviews, it is crucial to critically evaluate the methodological quality of existing systematic reviews before definitive evidence-based recommendations can be made.

The objective of this overview of systematic reviews was: (i) to evaluate and map the methodological quality of the available systematic reviews; (ii) to summarize the existing evidence on the effectiveness of mammography screening in reducing breast cancer mortality; and (iii) to summarize the evidence regarding the accuracy of different mammography techniques for breast cancer screening.

## Methods.

### Study Report

We reported this review according to the preferred reporting items for a systematic review and meta-analysis of diagnostic test accuracy (PRISMA-DTA) (McInnes et al. 2018).

### Eligibility criteria

The inclusion and exclusion criteria employed encompassed: (1) systematic reviews with meta-analysis that reported the value of mammography screening; (2) systematic reviews must include women who were enrolled in breast cancer screening programs without previously diagnosed breast cancer; (3) systematic reviews must report the value of screening mammography (association between mammography screening and breast cancer mortality reduction, sensitivity; specificity; cancer detection rate (CDR), area under curve (AUC), positive likelihood ratio (PLR), negative likelihood ratio (NLR), diagnostic odds ratio (DOR) and patient recall rate; (4) No language restrictions were applied. Studies were excluded if: (1) systematic reviews did not focus on mammography screening such as diagnosis and recurrence. (2) narrative/methodological reviews, books, protocols, conference abstracts, letters, and systematic reviews without meta-analysis.

### Search strategy

We performed a systematic search in Cochrane Library, EMBASE, and PubMed until Dec 20, 2022, to identify relevant systematic reviews. The databases were searched using following search terms: “breast neoplasm”, “phyllodes tumor”, “intraductal carcinoma”, “lobular carcinoma”, “mammography”, “systematic review”, and “meta-analysis". The detailed search strategies for each database are available in Supplement S1.

### Study selection

The identified records were imported into EndNote X20 (Thomson Reuters (Scientific) LLC Philadelphia, PA, US) for management. After the removal of duplicate records, the selection of potential reviews was performed first by titles and abstracts by two independent authors (JYS and YG). The records that did not meet the inclusion criteria were excluded. The full text of each potential review was then obtained and assessed by the same two reviewers (JYS and YG) to determine whether they meet the eligibility criteria. The discrepancies between the two authors were resolved by discussion with a third reviewer (JHT).

### Data extraction and management

All authors involved in this study piloted the data extraction form on a random sample of three included systematic reviews to ensure the agreement among the interpretation of data items. Reviewers (JYS, JL, and LZ) worked in pairs to ensure the accuracy of the data extraction process, one reviewer (JYS and JL) extracted data from the included studies using a data extraction sheet, and a second reviewer (ZL and JL) verified the extracted data. The extracted data included: authors, published year, country of the corresponding author, database searched, target population, intervention, intervention/exposure, comparison/control, outcome measures, study design, a methodological quality assessment tool used, number of included study and patients, follow up, the pooled results and their 95% confidence intervals (CI). Any disagreements were adjudicated by the third reviewer (JHT).

### Assessment of methodological quality

The ROBIS (Risk of Bias in Systematic Reviews) tool was used to assess the risk of bias in the included systematic reviews. This tool is applicable to various types of reviews, including diagnostic, etiological, prognostic, and interventional topics, aligning well with the diverse nature of the included studies. Its comprehensive evaluation ensures the reliability and relevance of the review findings (Perry et al. 2017; Whiting et al. 2016). All reviewers involved in this study piloted the ROBIS checklists form on a random sample of three included systematic reviews to ensure the agreement among each criterion of ROBIS prior to employing this tool. Two review authors (JYS, and JL) performed quality assessments independently using ROBIS, which is completed in three phases as follows: (1) Phase 1 aims to assess the alignment between the target problem and the issue addressed in the systematic review; (2) Phase 2 primarily assesses four domains of the systematic review: study eligibility criteria (5 items), identification and selection of studies (5 items), data collection and study appraisal (5 items), and synthesis and findings (6 items). If all signaling questions are "Yes" or "Probably Yes," the risk is "Low." If any response to the signaling questions is "No" or "Probably No," the risk of bias for that domain is deemed "High." If the provided information is insufficient to make a judgment, the degree of bias risk is "Uncertain"; (3) Phase 3 involves an overall risk of bias assessment for the systematic review by addressing three key signaling questions. This results in a final risk assessment of "Low," "High," or "Uncertain." Any disagreements were adjudicated by the third reviewer (JHT).

### Data synthesis

The pooled effect size estimates from the meta-analyses, along with their 95% confidence intervals (CIs), were expressed as odds ratios (OR), relative risks (RR), risk differences (RD), or event rates (ER), depending on the measures reported by the original authors. The characteristics of the included systematic reviews, the results of the risk of bias (RoB) assessment, and the pooled effect size estimates were summarized descriptively using systematically structured tables and evidence mapping.

## Results

### Search results

**Fig. 1:**
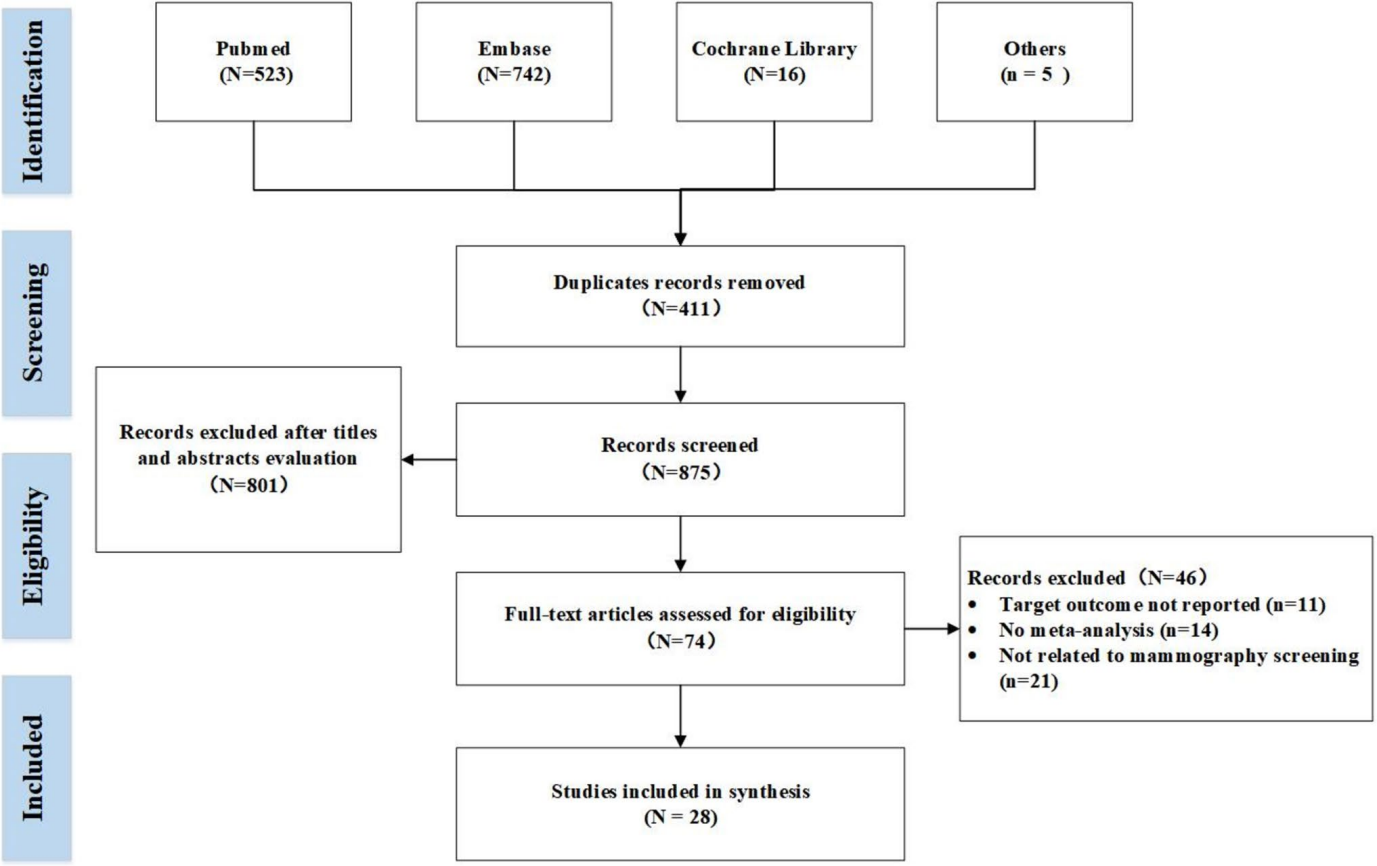
Flow diagram of the study selection

The search retrieved 1286 records. After removing duplicates, 411 articles were excluded. An additional 801 articles were excluded following the screening of titles and abstracts for obvious irrelevance. The remaining 74 records, 11 were excluded due to the lack of target outcomes of this review, 14 were excluded due to no meta-analysis conducted, 21 were excluded due to not focus on breast cancer screening. Ultimately, 28 studies were included in this review (Nelson et al. 2009; Zhu et al. 2018; Iared et al. 2011; Vinnicombe et al. 2009; Giampietro et al. 2020; Hodgson et al. 2016; Souza et al. 2013; Yun et al. 2017; Song et al. 2019; Marinovich et al. 2018; Phi et al. 2018, 2017; Farber et al. 2020; Nickson et al. 2012; Magnus et al. 2011; Gøtzsche and Olsen 2000; Hendrick et al. 1997; Kerlikowske et al. 1995; Jacklyn et al. 2016; Posso et al. 2017; Gøtzsche and Jørgensen 2013; Smart et al. 1995). Details of the PRISMA flow chart of literature studies for this overview are presented in Fig. 1.

### Characteristics of included systematic reviews

The detailed characteristics of the included 28 systematic reviews with meta-analysis included primary studies ranging from 4 to 24 are presented in Table 1, with 24 reviews from developed countries, 4 from developing countries. Twenty-four systematic reviews reported the databases searched, only 2 study retrieved Chinese databases. Sixteen reviews assessed the methodological quality, of which 13 used QUADAS-2, 1 used QUADAS, 1 used Cochrane RoB Assessment tool, and 1 used quality score evaluation system for assessing the quality of RCTs published by Chalmers et al.

**Table 1:** Main characteristics of included studies

Study	Country of corresponding author	PICO	Age	Follow up	Methodological quality assessment tool	Studies	Database searched
Abdullah (2021)	Canada	P:women without previously diagnosed breast cancer; I:DM, SM; C:SM + DBT, DM + DBT; O:sensitivity;specificity	No limination	NR	QUADAS-2	13	1)Medline; 2)Embase; 3)Cochrane Library
Zeng (2021)	China	P:average-risk patients eligible for breast cancer screening or in the diagnostic setting of having clinical suspicious findings for breast cancer; I:DBT, combined DBT and DM; C:DM alone; O:breast cancer mortality	over 40	NR	QUADAS-2	13	1) Embase; 2)Medline; 3)Web of Science; 4)Cochrane Library
Canelo-Aybar (2021)	Spain	P:women without previously diagnosed breast cancer; I:DBT, combined DBT and DM; C:DM alone; O:breast cancer mortality	No limination	NR	Cochrane RoB Assessment tool	10	1) PubMed; 2) Embase; 3) Cochrane Library
Dibden (2020)	UK	P:women without previously diagnosed breast cancer; I:breast cancer screening underwent mammography; C:women exposed to screening versus not screening; O:breast cancer mortality	No limination	NR	NR	27	1) PubMed
Farber (2020)	Australia	P:women without previously diagnosed breast cancer; I:FFDM; C:FM; O:CDR; Recall rate; FPR	No limination	NR	ROBINS-I	24	1) Embase; 2) Medline; 3) Web of Science; 4) Cochrane Library; 5) PubMed; 6) National Health Service Economic Evaluation Database; 7) National Health Service Economic Evaluation Database
Giampietro (2020)	Brazil	P:women, over 45 years of age and with no breast cancer related symptomsfrom among a population with a standard risk of developing breast cancer,who attended population-based breast cancer screenings; I:breast screen underwent digital breast tomosynthesis (DBT) + digital mammography(DM) and DBT + synthetic mammography C:accuracy and effectiveness of DBT, plusdigital or synthetic mammography, with digital mammography alone O:recall rate; CDR	Over 45	2 years	QUADAS-2	18	1) Embase; 2)P ubMed; 3) LILACS; 4) CENTRAL; 5) Trip Medical Database; 5) SCOPUS; 6) Web of Science; 7) CINAHL; 8) ClinicalTrials.gov
Farber (2020)	Australia	P:asymptomatic women who are at average risk of breast cancer;women at high risk of breast cancer were excluded; I:breast cancer screening underwent FFDM and FSM; C:women exposed to screening versus not screening; O:CDR;recall rates	No limination	1–2 years	ROBINS-I	24	1) Medline; 2) Embase; 3) PubMed; 4) Embase; 5) NHS Economic Evaluation Database; 6) Database of Abstracts of Reviews of Effects; 7) ACP Journal Club; 8) Cochrane Central Register of Controlled Trials; 9 Cochrane Library
Song (2019)	Korea	P:asymptomatic women aged 40 years or older who were enrolled in breast cancer screening programs or for whom mammography was recommended; I:breast screen underwent digital and screen-film mammography; C:performance of digital and screen-film mammography; O: sensitivity; specificity; AUC; DOR;CDR; NLR	over 40	1–2 years	QUADAS-2	13	1) Medline; 2) Embase; 3)Cochrane Library
Alabousi (2020)	Canada	P:average-risk patients eligible for breast cancer screening or in the diagnostic setting of having clinical suspicious findings for breast cancer; I:DBT, combined DBT and DM; C:DM alone; O:breast cancer mortality	No limination	NR	QUADAS-2	38	1) Medline; 2) Embase
Zhu (2018)	China	P:patients who underwent Contrast-Enhanced Spectral Mammography (CESM); I:intervention was CESM; C:histopathology or clinical follow-up results were comparison tests; O:sensitivity;specificity; diagnostic odds ratio (DOR); positive likelihood ratio(PLR); negative likelihood ratio(NLR). area under curve(AUC)	31 to 75	NR	QUADAS-2	18	1) PubMed; 2) the Cochrane library; 3) the China Biological Medicine Database; 4) China National Knowledge Infrastructure; 5) the VIP China Science and Technology Journal Database
Phi (2018)	Netherland	P:asymptomatic women older than 18 years, who underwent breast imaging using DBT and DM and were classified as having dense breasts on mammography; I:breast cancer screening and diagnosis underwent DBT and DM; C:women exposed to screening versus not screening; O:CDR;recall rate;sensitivity;specificity	over 18	NR	QUADAS-2	16	1) PubMed; 2) the Web of Science
Marinovich (2018)	Australia	P:asymptomatic women attending population breast cancer screening;symptomatic or high-risk women, or conducted in nonscreening settings (eg, assessment, diagnosis, staging) were ineligible; I:breast screen underwent DBT and 2D digital screening mammography; C:performance of DBT and 2D digital screening mammography; O:CDR;recall rate	53.8–59.5	NR	QUADAS-2	17	1) Embase; 2) Medline; 3) Database of Abstracts of Reviews of Effects; 4) Heath Technology Assessment; 5) NHS Economic Evaluation Database; 6) ACP Journal Club; 7) Cochrane Library
Yun (2017)	Korea	P:women participating in a breast cancer screening programme or who were undergoing opportunistic mammography screening; I:breast screen underwent DBT + FFDM and FFDM alone; C:performance of DBT + FFDM and FFDM alone; O:CDR	NA	NR	QUADAS-2	11	1) PubMed; 2) Embase; 3) Cochrane Library
Phi (2017)	The Netherlands	P:women aged 25 or older, who had a strong family history of breast cancer and no known gene mutation and had completed at least one screening round,women who were proven to be non-mutation carriers from a BRCA family were excluded; I:breast cancer screening underwent mammography C:women exposed to screening versus not screening; O:sensitivity;specificity	over 25	at least 1 year follow-up	NR	6	NR
Posso (2017)	Spain	P:women who underwent screening mammography; I:breast cancer screening underwent mammography C:women exposed to screening versus not screening; O:CDR;Recall rate	48–69	1 years	QUADAS-2	4	1) Medline; 2) Embase; 3) Cochrane Library
Jacklyn (2016)	Australia	P:average risk woman between the ages of 39 and 75 years who chooses to participate ‘regularly’ in screening; I: breast cancer screening underwent mammography; C:women exposed to screening versus not screening; O:breast cancer mortality	39–75	13 years	NR	9	NR
Nelson (2016)	USA	P:women without previously diagnosed breast cancer; I: breast cancer screening underwent mammography; C:women exposed to screening versus not screening; O:breast cancer mortality	over 40	NR	NR	16	1) PubMed; 2) Cochrane Library
Hodgson (2016)	UK	P:women participating in a breast cancer screening programme or who were undergoing opportunistic mammography screening; I:breast screen underwent DBT + FFDM and FFDM alone; C:performance of DBT + FFDM and FFDM alone; O:sensitivity;specificity	over 29.4	1 years	QUADAS-2	5	NR
Souza (2013)	Brazil	P:asymptomatic women under breast cancer screening; I:screen-film mammography and full-field digital mammography; C:accuracy of screen-film mammography and FFDM; O:sensitivity;specificity;PPV; PLR; AUC; DOR	NA	1–2 years	QUADAS	7	1) Medline; 2) Embase; 3) Cochrane Library; 4) Cochrane Database of Systematic Reviews; 4) Cochrane Central Register of Controlled Trials; 5) LILACS;6)SciELO; 7) EBM Review-Database of Abstracts of Review of Effects; 8) ACP Journal Club; 9) Cochrane Methodology Register; 10) NHSEED; 11) Journals@ OVID Full Text
Gøtzsche (2013)	Canada	P:women without previously diagnosed breast cancer.; I: breast cancer screening underwent mammography; C:women exposed to screening versus not screening; O:breast cancer mortality	39–74	13 years	NR	7	1) PubMed; 2) World Health Organization's International Clinical Trials Registry Platform
Nickson (2012)	Australia	P:women age range encompassing or overlapping substantially with our age group of interest (50 to 69 years); I:breast cancer screening underwent mammography; C:mortality benefit of screening for breast cancer; O:breast cancer mortality	40–75	NR	NR	10	1) Medline
Iared (2011)	Brazil	P:women age 40 or over who were enrolled in breast cancer screening programs or who complained of specific ailments, and for whom mammography was recommended; I:breast screen underwent digital mammography and film mammography (FM); C:performance of digital and film mammography; O:cancer detect rate (CDR); recall rate。	NR	NR	NR	11	1) PubMed; 2) Embase; 3) Lilacs; 4) Scopus
Magnus (2011)	USA	P:women 39–49 years of age at randomization, with follow-up exceeding 10 years; I:breast cancer screening underwent mammography; C:women exposed to screening versus not screening; O:breast cancer mortality	39–49	over 10 years (range 10.7–18)	quality score evaluation system published by Chalmers et al	7	1) PubMed; 2) Medline; 3) Cochrane Library; 4) Educational Resources Information Center
McCormack (2009)	UK	P:patients(50–70 years)who attend population-based screening program; I: breast screen underwent Full-Field Digital Screen and film mammography; C: performance of full-field digital mammography (FFDM); O: CDR;recall rate;positive predictive value(PPV)	over 50	NR	NR	8	1) PubMed; 2) Medline; 3) Embase
Gøtzsche (2000)	Denmark	P:women were excluded if breast cancer had been diagnosed before entry to the trial; I:breast cancer screening underwent mammography; C:women exposed to screening versus not screening; O:breast cancer mortality	39–59	9–12 years	NR	8	1) Cochrane Library
Hendrick (1997)	USA	P:women aged 40–49 at entry; I:breast cancer screening underwent mammography; C:women exposed to screening versus not screening; O:breast cancer mortality	40–49	10.5 to 18 years (average 12.7 years)	NR	8	NR
Kerlikowske (1995)	USA	P:women who underwent screening mammography; I:breast cancer screening underwent mammography; C:women exposed to screening versus not screening; O:breast cancer mortality	40–74	7–12 years	NR	13	1) Medline
Smart (1995)	USA	P:women ages 40 to 49 years; I:breast cancer screening underwent mammography; C:women exposed to screening versus not screening; O:breast cancer mortality	40–49	7–18 years	NR	8	NR

*CESM* Contrast-Enhanced Spectral Mammography, *DBT* digital breast tomosynthesis, *DOR* diagnostic odds ratio, *CDR* cancer detection rate, *AUC* area under curve, *PPV* positive predictive value, *DM* digital mammography, *FM* film mammography, *FPR* false-positive rate, *NLR* negative likelihood ratio

### Results of methodological quality

**Fig. 2:**
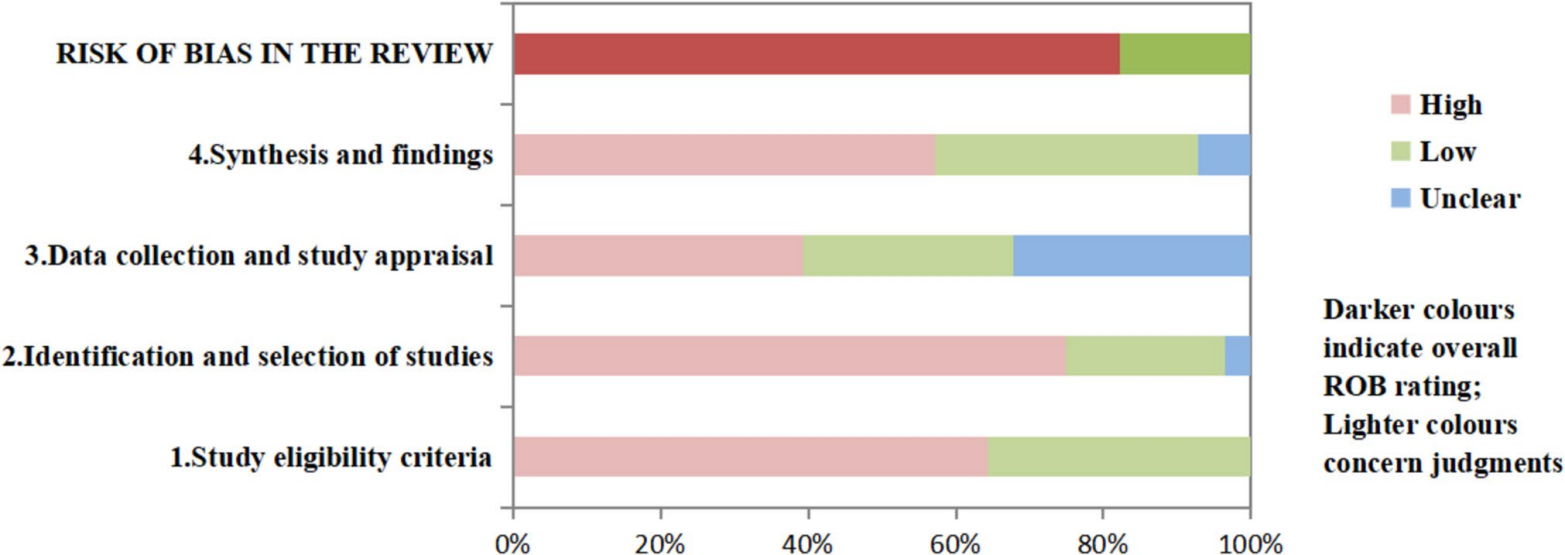
ROBIS result of 28 included studies

**Fig. 3:**
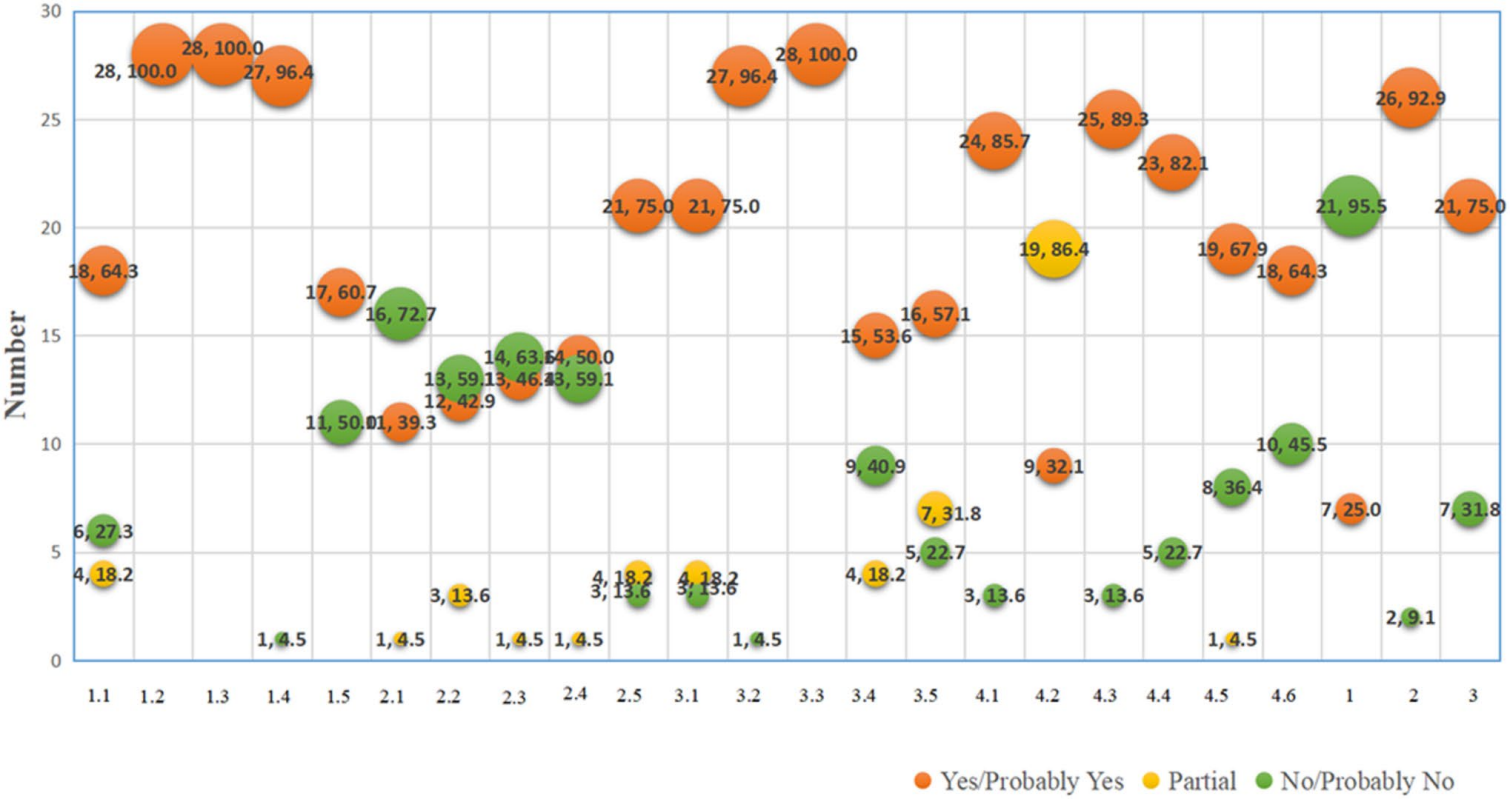
The full compliance rate of each ROBIS item

Methodological quality using the ROBIS tool was assessed in the 28 systematic reviews. Among the reviews, 18 systematic reviews (64.3%) were classified as having a high risk of bias (RoBs) in Domain 1: Study Eligibility Criteria. In Domain 2: Identification and Selection of Studies, 75% (21 systematic reviews) were rated with high RoBs. A total of 9 systematic reviews (32.1%) were considered to have high RoBs in Domain 3: Data Collection and Study Characteristics. Domain 4: Statistical Analysis and Interpretation showed that 57.1% (16 systematic reviews) had high RoBs. When considering the overall risk of bias, 23 systematic reviews (82.1%) were judged to have high RoBs. The complete ROBIS assessment results are illustrated in Figs. 2 and 3.

In terms of study eligibility criteria, 36.4% of the systematic reviews were assessed with low RoBs. Notably, only nine systematic reviews (32.1%) reported prior study protocols or registration, consistent with previous findings. For Domain 2 (Identification and Selection of Studies), 18.2% of the systematic reviews demonstrated low risk for each signaling question. Sixteen systematic reviews conducted searches across three or more databases, while twelve reported a detailed search strategy, identified unpublished studies, and searched registry platforms. Eight studies did not report clear follow-up times, and seven did not involve at least two reviewers independently performing the process of selecting studies for inclusion. Half of the systematic reviews used appropriate tools to assess risk of bias or methodological quality, and only eight studies assessed the RoBs of included systematic reviews independently with at least two reviewers.

### Association between mammography screening and breast cancer mortality

Eleven systematic reviews compared the association of mammography vs. no screening on breast cancer mortality. Pooled estimates for reduction in breast cancer mortality attributable to mammography screening stratified by study design ranged from 0.51 (OR, 95% CI 0.46–0.55) to 1.04 (RR, 95% CI 0.84–1.27). An systematic reviews of 10 case–control studies average a 49% reduction in breast cancer mortality for women who are screened (0.51 [OR, 95% CI 0.46–0.55]), which was similar to systematic reviews with RCTs conducted by Gøtzsche et al. (0.75 [RR, 95% CI 0.67–0.83]), Hendrick et al. (0.71 [RR, 95% CI 0.57–0.89] and Cochrane analysis (0.71 [RR, 95% CI 0.61–0.83]) (Gøtzsche and Olsen 2000; Hendrick et al. 1997; Gøtzsche and Jørgensen 2013). Pooled estimates of systematic reviews with RCTs only conducted by Magnus (0.83 [RR, 95% CI 0.72–0.97]), Gøtzschewere et al. (1.04 [RR, 95% CI 0.84–1.27]), and Cochrane analysis (0.93 [RR, 95% CI 0.79–1.09]) generally higher (mortality reduction lower) than with those observed with the other study design trials (Table 2) (Magnus et al. 2011; Gøtzsche and Olsen 2000; Gøtzsche and Jørgensen 2013).

**Table 2:** Main results of the included meta-analysis evaluating association between mammography screening and breast cancer mortality

Study	Age	Study design	Participates	Follow up	ES (95% CI)
Canelo-Aybar (2021)	50–69	RCT(10)	348478	3.5–18 years	RR:0.77 (95% CI 0.66,0.90)
	70–74	RCT(10)	249930	3.5–18 years	RR:0.88 (95% CI 0.76,1.02)
Dibden (2020)	35–74	CS(27)	NR	1.5–3 years	RR:0.78 (95% CI 0.75,1.82)
Nelson (2016)	39–49	RCTs (9)	NR	11.2–21.9 years	RR:0.92 (95% CI 0.75,1.02)
	50–59	RCTs (7)	NR	12.5–21.9 years	RR:0.86 (95% CI 0.68,0.97)
	69–69	RCTs (5)	NR	12.5–15.5 years	RR:0.67 (95% CI 0.54,0.83)
	70–74	RCTs (3)	NR	12.5–13.6 years	RR:0.80 (95% CI 0.51,1.28)
Jacklyn (2016)	39–75	RCTs(9)	599090	13 years	RR:0.78 (95% CI 0.72,0.85)
Gøtzsche (2013)	39–74	ART (4) SRT (7)	616327	7 years	RR:0.81 (95% CI 0.72,0.90)
		ART (4)	292958	7 years	RR:0.93 (95% CI 0.79,1.09)
		SRT (7)	323369	7 years	RR:0.71 (95% CI 0.61,0.83)
		ART (4) SRT (5)	599090	13 years	RR:0.81 (95% CI 0.74,0.87)
		ART (4)	292153	13 years	RR:0.90 (95% CI 0.79,1.02)
		SRT (5)	306937	13 years	RR:0.75 (95% CI 0.67,0.83)
	< 50	ART (3) SRT (5)	356368	7 years	RR:0.89 (95% CI 0.77,1.04)
		ART (3)	227333	7 years	RR:0.94 (95% CI 0.78,1.14)
		SRT (5)	129035	7 years	RR:0.81 (95% CI 0.63,1.05)
		ART (3) SRT (5)	329511	13 years	RR:0.84 (95% CI 0.73,0.96)
		ART (3)	218697	13 years	RR:0.87 (95% CI 0.73,1.03)
		SRT (5)	110,814	13 years	RR:0.80 (95% CI 0.64,0.98)
	≥ 50	ART (2) SRT (5)	261044	7 years	RR:0.72 (95% CI 0.62,0.85)
		ART (2)	65625	7 years	RR:0.88 (95% CI 0.64,1.20)
		SRT (5)	195419	7 years	RR:0.94 (95% CI 0.77,1.15)
		ART(2) SRT (5)	268874	13 years	RR:0.77 (95% CI 0.69,0.86)
		ART (2)	74261	13 years	RR:0.94 (95% CI 0.77,1.15)
		SRT (5)	194613	13 years	RR:0.70 (95% CI 0.62,0.80)
Nickson (2012)	40–75	CS(10)	NR	NR	OR:0.51 (95% CI 0.46,0.55)
Magnus (2011)	39–49	RCTs(7)	336261	10–18 years	RR:0.83 (95% CI 0.72,0.97)
Gøtzsche (2000)	39–59	RCTs(2)	132118	9–12 year	RR:1.04 (95% CI 0.84,1.27)
		quasi-RCTs(6)	324231	9–12 year	RR:0.75 (95% CI 0.67,0.83)
Hendrick (1997)	40–49	RCTs(8)	211150	12.7 years(rang from 10.5 to 18)	RR:0.82 (95% CI 0.71,0.95)
Kerlikowske (1995)	40–49	RCTs(8);CS(1)	NR	7–12 years	RR:0.93 (95% CI 0.76,1.13)
		RCTs(8);CS(1)	NR	7–9 years	RR:1.02 (95% CI 0.82,1.27)
		RCTs(8);CS(1)	NR	10–12 years	RR:0.83 (95% CI 0.65,1.06)
	40–74	RCTs(8);CS(4)	NR	7–12 years	RR:0.75 (95% CI 0.68,0.83)
		RCTs(8);CS(5)	NR	7–9 years	RR:0.78 (95% CI 0.69,0.89)
		RCTs(8);CS(6)	NR	10–12 years	RR:0.77 (95% CI 0.68,0.86)
	50–74	RCTs(8);CS(2)	NR	7–12 years	RR:0.74 (95% CI 0.66,0.83)
		RCTs(8);CS(2)	NR	7–9 years	RR:0.73 (95% CI 0.63,0.84)
		RCTs(8);CS(2)	NR	10–12 years	RR:0.76 (95% CI 0.67,0.87)
Charles (1995)	40–49	RCTs (7)	128,519	7 years	RR:0.76 (95% CI 0.62,0.95)

*RCT* randomised controlled trial, *CS* cohort studies, *ART* adequately randomised trials, *SRT* suboptimally randomised trials

In the systematic reviews of RCTs that stratified by age, a significantly reduced breast cancer mortality among women with the latest follow-up data (aged 40–49) invited to screening mammography was observed by Hendrick et al. (0.82[RR, 95% CI 0.71–0.95], 7 RCTs) and Magnus et al. (0.83 [RR, 95% CI 0.72–0.97)], 8 RCTs) (Magnus et al. 2011 Jun; Hendrick et al. 1997); however, pooled estimates are not statistically significant for systematic reviews conducted by Nelson et al. (0.92, [RR, 95% CI 0.75–1.02], 9 trials), and Smart et al. (0.84 [RR, 95% CI 0.69–1.02], 8 RCTs) (Nelson et al. 2009; Smart et al. 1995). The Cochrane analysis with adequately randomized trials found no significant breast cancer mortality reduction among women below 50 years for follow up after 7 years (0.94 [RR, 95% CI 0.78–1.14]) and 13 years (0.87 [RR, 95% CI 0.73–1.03]). The Cochrane analysis reported that the pooled estimates for mortality reduction in screening women aged 50 years or older was lower when combined sub-optimally randomized trials with 7 and 13 years follow up (0.88 [RR, 95% CI 0.64–1.20] and 0.94 [RR, 95% CI 0.77–1.15]) than adequately randomized trials with 7 and 13 years (0.67 [RR, 95% CI 0.56–0.81] and 0.70 [RR, 95% CI 0.62–0.80]) (Gøtzsche and Jørgensen 2013). An systematic reviews conducted by Nelson et al. observed a statistically significant difference among women aged 50 to 59 (0.86 [RR, 95% CI 0.68–0.97]) and aged 60–69 years (0.67 [RR, 95% CI 0.54–0.83]), the combined RR is not statistically significant for women aged 70–74 (P > 0.05) (Nelson et al. 2009). Canelo-Aybar et al. found high certainty evidence that mammography screening reduces breast cancer mortality risk for women 50–69 (0.77 [RR, 95% CI 0.66–0.90]).

### Performance of mammography screening technology

Nine systematic reviews reported the diagnostic accuracy of breast cancer screening conducted using mammography (Table 3). Sensitivity of different types of mammography was ranged from 55% to 90.77%, specificity of different types of mammography ranged from 84% to 97%. The sensitivity of digital breast tomosynthesis + full-field digital mammography, contrast-enhanced spectral mammography, digital mammography, film mammography, and full-field digital mammography, were 90.77% (95% CI 80.7–96.51%), 89% (95% CI 88–91%), 76% (95% CI 70–81%), 76% (95% CI 70–81%), and 60.00% (95% CI 47.10–71.96%), respectively. The specificity of film mammography, digital breast tomosynthesis + full-field digital mammography, digital mammography, full-field digital mammography, and contrast-enhanced spectral mammography were 97% (95% CI 94–98%), 96.49% (95% CI 96.04–96.90%), 96% (95% CI 94–97%), 95.55% (95% CI 95.04–96.01%), and 84% (95% CI 82–85%). The DOR of breast cancer screening conducted using the contrast-enhanced spectral mammography, digital mammography, and film mammography were 71.36(95% CI 36.28–140.39), 72(95% CI 44–118), and 91(95% CI 52–157). However, Alabousi et al. found that synthetic 2D mammography and synthetic 2D mammography plus digital breast tomosynthesis showed comparable diagnostic accuracy to digital mammography and digital mammography plus digital breast tomosynthesis, respectively.

**Table 3:** Main results and subgroup analysis of the included meta-analysis evaluating the accuracy of screening mammography

No.study	Intervention	Sensitivity (95% CI)	Specificity (95% CI)	AUC (95% CI)	PLR (95% CI)	NLR (95% CI)	DOR (95% CI)
Alabousi (2020)	DBT + DM	84% (80%, 88%)	91% (83%, 95%)	0.91 (0.88,0.93)			
	DM	73% (65%, 80%)	88% (77%, 94%)	0.85 (0.81,0.88)			
	SM	75% (67%, 82%)	92% (85%, 96%)	0.88 (0.85,0.91)			
	DBT + SM	85% (80%, 89%)	93% (86%, 96%)	0.92 (0.90,0.94)			
Song (2019)	DM	76% (70%, 81%)	96% (94%, 97%)	0.94 (0.92,0.96)	18.0 (12.2,26.7)	0.25 (0.20,0.32)	72 (44,118)
	FM	76% (70%, 81%)	97% (94%, 98%)	0.92 (0.89,0.94)	22.5 (13.1,38.8)	0.25 (0.20,0.31)	91 (52,157)
Song (2019)	Mammography	85.7% (77.7%, 92.2%)	95.3% (CI 95% 94.4%, 96.1%)	NR	NR	NR	NR
Zhu (2018)	CESM	89% (88%, 91%)	84% (82%, 85%)	0.96 (0.94,0.98)	3.73 (2.68,5.2)	0.1 (0.06,0.15)	71.36 (36.28,140.39)
Phi (2017)	Mammography	55% (41%, 69%)	94% (90%, 96%)	NR	NR	NR	NR
Hodgson (2016)	DBT + FFDM	90.77% (80.7%, 96.51%)	96.49% (96.04%, 96.90%)	NR	NR	NR	NR
	FFDM	60.00% (47.10%, 71.96%)	95.55% (95.04%, 96.01%)	NR	NR	NR	NR
Souza (2013)	FM	NR	NR	0.92 (0.91, 0.92)	NR	NR	NR
	FFDM	NR	NR	0.91 (0.89,0.93)	NR	NR	NR

*AUC* area under curve, *PLR* Positive likelihood Ratio, *NLR* Negative likelihood ratio, *DOR* diagnostic odds ratio

Seven systematic reviews compared the recall rate and CDR of screening mammography of different mammography (Table 4). The pooled estimates for CDR of digital breast tomosynthesis vs. digital mammography, digital breast tomosynthesis + synthetic 2D mammography vs. digital mammography, and digital breast tomosynthesis + full-field digital mammography vs. full-field digital mammography were 0.0016 (RD, 95% CI 0.0011–0.002), 1.38(RR, 95% CI 1.24–1.54), and 1.29 (RR, 95% CI 1.164–1.429). The CDR of film mammography was similar to full-field digital mammography (0.93[RR, 95% CI 0.83–1.03]), two studies evaluated the CDR of digital breast tomosynthesis + digital mammography and digital mammography and statistically significant were both observed (1.36 [RR, 95% CI 1.18–1.58] and 1.52 [RR, 95% CI 1.08–2.12]). Moreover, the pooled estimates for CDR of digital mammography vs. film mammography in two systematic reviews found a statistically significant (1.17 [RR, 95% CI 1.06–1.29] and 0.00051 [RD, 95% CI 0.00019–0.00083]). The risk ratios and the respective 95% CI of recall rate of digital mammography vs. film mammography in two systematic reviews were inconsistent (1.07 [RR, 95% CI 0.94–1.22] and 0.00695 [RD, 95% CI 0.00347–0.01042]). Moreover, the risk ratios and the respective 95% CI of recall rate of digital breast tomosynthesis + digital mammography vs. digital mammography were also inconsistent (1.13 [RR, 95% CI 0.96–1.32] and 0.72[RR, 95% CI 0.64–0.80]). The recall rate of film mammography vs. full-field digital mammography and digital breast tomosynthesis + synthetic 2D mammography vs. digital mammography were not statistically significant (0.95 [RR, 95% CI 0.71–1.26] and 1.08 [RR, 95% CI 0.92–1.26]). The RD of recall rate of digital breast tomosynthesis was statistically significant lower than digital mammography (− 0.0219 [− 0.0298, − 0.014]). Screening using synthetic 2D mammography + digital breast tomosynthesis has similar breast cancer detection but reduces recall and biopsy when compared with digital mammography + digital breast tomosynthesis.

**Table 4:** Main results and subgroup analysis of the included meta-analysis evaluating the recall rate and CDR of screening mammography

No. study	Intervention (A)	Intervention (B)	Recall rate			CDR		
			Recall rate (A)	CDR (A)	CDR (B)	ES(95% CI) (A VS B)	Recall rate (B)	ES(95% CI) (A VS B)
Zeng (2021)	SM + DBT	DM + DBT	4.4/1000	7.2/1000	6.3/1000	RD:− 0.0001 (− 0.4,0.2)	6.0/1000	RD:− 0.56 (− 1.03,− 0.08)
	DM	SM + DBT	5/1000	5.5/1000	7.2/1000	RD:− 0.0095 (− 0.0191,− 0.0001)	4.4/1000	RD:2.0/1000 (1.4,2.6)
Farber (2020)	DM	FM	119638/386799238	7874/4477071	15479/8571434	− 0.0000 2(− 0.00006,0.00003)	261652/8110599	RD:− 0.95 (− 1.91,− 0.002)
Farber (2020)	DM	FM	119638/3867923	31015/5614900	56218/10968843	RD: 0.00051 (0.00019,0.00083)	261652/8110599	RD: 0.00695 (0.00347, 0.01042)
Giampietro (2020)	DBT + DM	DM	25834/341780	474/48482	347/48488	RR: 1.36 (1.18,1.58)	29715/437716	RR: 1.36 (1.18,1.58)
	DBT + SM	DM	3196/94578	754/94568	702/119359	RR: 1.38 (1.24,1.54)	3905/119359	RR: 1.08 (0.92,1.26)
Marinovich (2018)	DBT	DM	31337/381424	2093/350810	2939/658980	RD: 0.0016 (0.0011,0.002)	69626/678826	RD:− 0.0219 (− 0.0298,− 0.014)
Phi (2018)	DBT + DM	DM	12705/12221	704/115838	854/188419	RR: 1.52 (1.08,2.12)	27808/226967	RR: 0.72 (0.64,0.8)
Yun (2017)	DBT + FFDM	FFDM	743/112624	NA	NA	NA	970/212917	RR: 1.29 (1.164,1.429)
Iared (2011)	DM	FM	10497/190322	1024/190,322	3599/638348	RR: 1.17 (1.06, 1.29)	26745/638348	RR: 1.07 (0.94, 1.22)
Vinnicombe (2009)	FM	FFDM	1404/31720	205/31720	58/8478	RR: 0.93 (0.83,1.03)	406/8478	RR: 0.95 (0.71,1.26)

*ES* effect size, *CDR* cancer detection rate

## Discussion

To our knowledge, this overview is the first one to systematically evaluate the methodological quality of evidence from systematic reviews to provide an evidence-based assessment and summarize the accuracy and efficacy on breast cancer screen. A comprehensive literature search was performed and 28 systematic reviews were identified, the number of studies included in each systematic reviews ranged from 4 to 24.

### Risk of bias in systematic reviews

This study reveals some methodological weaknesses in included systematic reviews, with only 17.9% meeting the ROBIS tool’s low-risk criteria. Over 60% of the systematic reviews exhibited deficiencies in defining study eligibility criteria, primarily due to unclear or inadequate reporting and the absence of pre-established protocols. Only a few SR reported using a protocol, the lack of a clear protocol increases the risk of selective reporting and other biases, undermining the reliability of the review’s conclusions. Additionally, many systematic reviews were limited by narrow database searches, failed to include grey literature, and lacked detailed search strategies, further heightening the potential for bias and making replication difficult. The absence of independent reviewers in the selection and evaluation process, a fundamental safeguard against bias, was also a common issue. Moreover, some studies included both cohort and RCT designs, which could introduce bias due to the differing nature of these study designs. This highlights the importance of proper risk of bias assessment and evidence quality evaluation. Unfortunately, many reviews failed to adequately report the risk of bias assessments and evidence quality, further compromising the reliability of their findings.

Future research in this area should consider the use of appropriate subgroup analyses or meta-regression techniques to address the observed heterogeneity in results. Significant differences in outcome indicators were found across studies, and a core set of indicators could be established in the future to standardize reporting and improve consistency. The findings underscore the urgent need for improved rigor, transparency, and consistency in the design, reporting, and execution of systematic reviews to enhance their credibility and relevance in healthcare practice.

### Association between mammography screening and breast cancer mortality

Nine systematic reviews explored the effectiveness of mammography screening, pooled estimates for the reduction in breast cancer mortality were ranging from 0.51 to 1.04 (OR or RR). The role of mammography-targeted screening in reducing mortality varies across studies, and this inconsistency may be influenced by several factors, including whether the study is a fully randomized trial, the overall study quality, statistical measures (such as OR or RR), and the age groups included. Specifically, in non-randomized trials, the use of OR tends to overestimate the effect size, especially when the outcome (breast cancer mortality) is not rare, and biases introduced by the study design can further distort the true effectiveness of screening.

In the systematic reviews that were stratified by age, although screening mammography has been shown to reduce breast cancer mortality among women aged 40–49 years, some studies did not achieve statistical significance. For women aged 50–69 years, systematic reviews consistently indicate that screening significantly decreases breast cancer mortality, particularly in high-quality randomized trials where the effect is more pronounced. In contrast, stratified results for women aged 70–74 years did not demonstrate a significant reduction in mortality, potentially due to competing mortality risks and decreased screening sensitivity. It is worth noting that non-randomized trials may exaggerate the effectiveness of breast cancer screening. Future high-quality randomized studies are necessary to define the value of screening across different age groups, aiming to maximize benefits while minimizing potential risks.

### Performance of mammography screening technology

Improvements in mammographic technology provided the transition from film mammography to digital mammography and digital breast tomosynthesis, which may increase the benefit of screening by increasing the detection accuracy of breast cancers. Five studies reported the sensitivity and specificity of mammography ranged from 55% to 90.77% and 84% to 97%. Hodgson et al. found digital breast tomosynthesis plus full-field digital mammography has higher sensitivity and specificity than full-field digital mammography alone, and Song et al. observed that the performance of digital mammography was similar to that of film mammography (Hodgson et al. 2016; Song et al. 2019). Zhu et al. found that contrast-enhanced spectral mammography has high diagnostic accuracy, while significant heterogeneity was observed and no potential source of heterogeneity could analysis due to partial loss of data.

Six studies compared the CDR and recall rate of different mammography techniques. Both systematic reviews conducted by Iared et al. and Farber et al. reported that the CDR of digital mammography was statistically significantly higher than that of film mammography. However, while Iared et al. found no significant difference in recall rates, Farber et al. observed a statistically significant increase (Iared et al. 2011; Farber et al. 2020). Conversely, Vinnicombe et al. reported no evidence of differences in CDR or recall rates between full-field digital mammography and film mammography (Vinnicombe et al. 2009). Marinovich et al. demonstrated statistically significant improvements in CDR and a reduction in recall rates when comparing digital breast tomosynthesis with digital mammography. The use of digital breast tomosynthesis combined with digital or synthetic mammography has been shown to improve the CDR for breast cancer. Phi et al. reported a reduction in the recall rate with this combination, whereas Giampietro et al. found no significant improvement in recall rate (Giampietro et al. 2020; Phi et al. 2018). Yun et al. demonstrated that adding digital breast tomosynthesis to full-field digital mammography is more effective in detecting breast cancer compared to full-field digital mammography alone, particularly in increasing the detection of early invasive breast cancers and improving specificity and CDR, compared to digital breast tomosynthesis alone (Yun et al. 2017). According to the results of the included systematic reviews, most reviews suggested that digital breast tomosynthesis increases the CDR and reduces the recall rate compared to digital mammography, while digital mammography increases the CDR compared to film mammography. Additionally, combining digital breast tomosynthesis with digital or synthetic mammography improves sensitivity, specificity, and CDR compared to digital breast tomosynthesis alone. However, the interpretation of these findings is challenged by potential methodological flaws and variations in reference standards. Our previous study also highlighted that differing guideline grading systems may limit the promotion and implementation of evidence (Cai et al. 2021). Therefore, more high-quality systematic reviews and primary studies are necessary to comprehensively assess the performance of different mammography techniques. It is also important to note the substantial variability in the selection of reported outcomes for evaluating the accuracy of mammography screening across studies, making it difficult to compare the effectiveness of different mammography methods.

### Strengths and limitations

To our knowledge, this is the first overview to investigate the methodological quality of systematic reviews using the ROBIS tool and to assess the benefits of mammography screening in reducing breast cancer mortality and improving the accuracy of mammography. However, the present overview has several limitations: First, the systematic reviews included in this overview were published between 1995 and 2022. The observed reduction in breast cancer mortality may be influenced by improvements in the treatment of advanced cancer, increased awareness of breast cancer, and other evolving factors. Additionally, the decision to participate in screening programs involves a careful consideration of trade-offs, including over-diagnosis, costs, and other influencing factors. Secondly, we only searched English databases, and although there were no language restrictions during the search, we ultimately did not find research in other languages, which may have missed studies in other language databases (such as the Chinese database CNKI). Finally, the high risk of bias (RoB) in some of the included systematic reviews may affect the reliability of the evidence synthesized.

## Conclusion

Most of the included reviews concluded that breast cancer mortality is generally reduced with mammography screening, however, there are still differences in the effects of different age groups. With the development of advanced imaging technology, the diagnostic performance of mammography has been improving, compared with digital mammography alone, synthetic 2D mammography alone, digital breast tomosynthesis alone, digital breast tomosynthesis plus synthetic 2D mammography or digital mammography depicted more cancers and reduced false-positive findings, but the level of evidence was low due to the differences in definitions, populations and the lack of comprehensive comparison of various intervention measures. Overall, the methodological quality of most of the included reviews was defined as high risk, with the publication of methodological tool, as well as reporting checklist, improvement is urgent need over time.

## Supplementary Information

Below is the link to the electronic supplementary material.

Supplementary file 1 (DOCX 13 KB)

## Data Availability

No datasets were generated or analysed during the current study.

## Abbreviations

SM: Synthetic 2D mammography
DM: Digital mammography
CESM: Contrast-enhanced spectral mammography
DBT: Digital breast tomosynthesis
FM: Film mammography
FFDM: Full-field digital mammography
CBM: Chinese biomedical databases
ROBIS: The risk of bias in systematic reviews
DOR: Diagnostic odds ratio
CDR: Cancer detection rate
AUC: Area under curve
PPV: Positive predictive value
FPR: False-positive rate
NLR: Negative likelihood ratio
RCT: Randomised controlled trial
