# Supplementary material for: The screening value of mammography for breast cancer: an overview of 28 systematic reviews with evidence mapping
Source: J Cancer Res Clin Oncol. 2025 Mar 6;151(3):102. doi: 10.1007/s00432-025-06122-z (PMC11885354; doi:10.1007/s00432-025-06122-z)
Supplement: Supplementary file 1 — (DOCX 13 KB) [file 432_2025_6122_MOESM1_ESM.docx]

**Table 1 Search strategy of PubMed database**

| #1 "Breast Neoplasms"[Mesh] OR "Breast Carcinoma In Situ"[Mesh] OR "Breast Neoplasms, Male"[Mesh] OR "Carcinoma, Ductal, Breast"[Mesh] OR "Carcinoma, Lobular"[Mesh] OR "Inflammatory Breast Neoplasms"[Mesh] OR "Triple Negative Breast Neoplasms"[Mesh] OR "Unilateral Breast Neoplasms"[Mesh]  #2 "Breast Neoplasm*"[Title/Abstract] OR "Breast Tumor*"[Title/Abstract] Or "Breast Carcinoma*"[Title/Abstract] OR "Breast Cancer*"[Title/Abstract] OR "Breast Tumour*"[Title/Abstract] OR "Mammary Neoplasm*"[Title/Abstract] OR "Mammary Tumor*"[Title/Abstract] OR "Mammary Carcinoma*"[Title/Abstract] OR "Mammary Cancer*"[Title/Abstract] OR "Mammary Tumour*"[Title/Abstract] OR "Breast Adenocarcinoma*"[Title/Abstract] OR "Breast Carcinogenesis"[Title/Abstract] OR "Breast Sarcoma*"[Title/Abstract] OR "Phyllodes Tumor*"[Title/Abstract] OR "Intraductal Carcinoma*"[Title/Abstract] OR "Lobular Carcinoma*"[Title/Abstract]  #3 #1 OR #2  #4 "Mammography"[Mesh]  #5 Mammograph*[Title/Abstract] OR "Digital Mammography"[Title/Abstract] OR "Digital Mammograph*"[Title/Abstract] OR "Digital Breast Tomosynthes*"[Title/Abstract] OR 3D-Mammograph*[Title/Abstract] OR 3D Mammography[Title/Abstract] OR "X-ray Breast Tomosynthes*"[Title/Abstract] OR "X-ray Breast Tomosynthes*"[Title/Abstract]  #6 #4 OR #5  #7 "Meta-Analysis" [Publication Type] OR "Meta-Analysis as Topic"[Mesh]  #8 Meta-Analysis[Title/Abstract] OR "Meta Analysis"[Title/Abstract] OR Meta-Analyses[Title/Abstract] OR "Meta Analyses"[Title/Abstract] OR "Gathering Analysis"[Title/Abstract]  #9 "Systematic evaluation"[Title/Abstract] OR "Systematic assessment" [Title/Abstract] OR "Systematic review*"[Title/Abstract] OR "System evaluation" [Title/Abstract] OR "System Assessment"[Title/Abstract] OR "Systemic Review*" [Title/Abstract]  #10 #7 OR #8 OR #9  #11 #3 AND #6 AND #10 |
| --- |

**Search strategy of EMBASE database**

| #1 'Breast Neoplasms'/exp OR 'Breast Carcinoma in Situ'/exp OR 'Breast Neoplasms, Male'/exp OR 'Ductal Breast Carcinoma'/exp OR 'Lobular Breast Carcinoma'/exp OR 'Inflammatory Breast Neoplasms'/exp OR 'Triple Negative Breast Neoplasms'/exp OR 'Unilateral Breast Neoplasms'/exp  #2 'Breast Neoplasm*':ti,ab OR 'Breast Tumor*':ti,ab OR 'Breast Carcinoma*':ti,ab OR 'Breast Cancer*':ti,ab OR 'Breast Tumour*':ti,ab OR 'Mammary Neoplasm*':ti,ab OR 'Mammary Tumor*':ti,ab OR 'Mammary Carcinoma*':ti,ab OR 'Mammary Cancer*':ti,ab OR 'Mammary Tumour*':ti,ab OR 'Breast Adenocarcinoma*':ti,ab OR 'Breast Carcinogenesis':ti,ab OR 'Breast Sarcoma*':ti,ab OR 'Phyllodes Tumor*':ti,ab OR 'Intraductal Carcinoma*':ti,ab OR 'Lobular Carcinoma*':ti,ab  #3 #1 OR #2  #4 MeSH descriptor: Diagnostic Imaging Explode all trees  #5 'Mammograph*':ti,ab OR 'Digital Mammography':ti,ab OR 'Digital Mammograph*':ti,ab OR 'Digital Breast Tomosynthesis*':ti,ab OR '3D Mammograph*':ti,ab OR '3D Mammography':ti,ab OR 'X-ray Breast Tomosynthesis*':ti,ab  #6 #4 OR #5  #7 'Meta-Analysis'/exp OR 'Meta-Analysis as Topic'/exp  #8 'Meta-Analysis':ti,ab OR 'Meta Analysis':ti,ab OR 'Meta-Analyses':ti,ab OR 'Meta Analyses':ti,ab OR 'Gathering Analysis':ti,ab OR 'Systematic review*':ti,ab OR 'Systemic Review*':ti,ab  #9 #7 OR #8  #10 #3 AND #6 AND #9 |
| --- |

**Search strategy of Cochrane database**

| #1 MeSH descriptor: [Breast Neoplasms] explode all trees  #2 "Breast Neoplasm" OR "Breast Neoplasms" OR "Breast Tumor" OR "Breast Tumors" OR "Breast Carcinoma" OR "Breast Carcinomas" OR "Breast Cancer" OR "Breast Cancers" OR "Breast Tumour" OR "Breast Tumours" OR "Mammary Neoplasm" OR "Mammary Neoplasms" OR "Mammary Tumor" OR "Mammary Tumors" OR "Mammary Carcinoma" OR "Mammary Carcinomas" OR "Mammary Cancer" OR "Mammary Cancers" OR "Mammary Tumour" OR "Mammary Tumours" OR "Breast Adenocarcinoma" OR "Breast Adenocarcinomas" OR "Breast Carcinogenesis" OR "Breast Sarcoma" OR "Breast Sarcomas" OR "Phyllodes Tumor" OR "Phyllodes Tumors" OR "Intraductal Carcinoma" OR "Intraductal Carcinomas" OR "Lobular Carcinoma" OR "Lobular Carcinomas"  #3 #1 OR #2  #4 MeSH descriptor: [Diagnosis] explode all trees  #5 "Mammograph" OR "Mammographs" OR "Digital Mammography" OR "Digital Mammographs" OR "Digital Breast Tomosynthesis" OR "3D Mammograph" OR "3D Mammographs" OR "3D Mammography" OR "3D Mammographies" OR "X-ray Breast Tomosynthesis" OR "X-ray Breast Tomosyntheses"  #6 #4 OR #5  #7 #3 AND #6 |
| --- |
